# Cytotoxic properties of the anthraquinone derivatives isolated from the roots of *Rubia philippinensis*

**DOI:** 10.1186/s12906-018-2253-2

**Published:** 2018-07-03

**Authors:** Vivek K. Bajpai, Md Badrul Alam, Khong Trong Quan, Hee-Jeong Choi, Hongyan An, Mi-Kyoung Ju, Sang-Han Lee, Yun Suk Huh, Young-Kyu Han, MinKyun Na

**Affiliations:** 1 Department of Energy and Materials Engineering, Dongguk University-Seoul, Seoul, 04620 Republic of Korea; 2 Department of Food Science and Biotechnology, Graduate School, Kyungpook National University, Daegu, 41566 Republic of Korea; 3 Food and Bio-Industry Research Institute, Kyungpook National University, Daegu, 41566 Republic of Korea; 4 College of Pharmacy, Chungnam National University, Daejeon, 34134 Republic of Korea; 5 Department of Biological Engineering, Biohybrid Systems Research Center (BSRC), Inha University, 100 Inha-ro, Nam-gu, Incheon, 22212 Republic of Korea

**Keywords:** *Rubia philippinensis*, Anthraquinone, Cytotoxicity, Breast cancer, Skin cancer

## Abstract

**Background:**

Cancer is one of the most frequently occurring diseases and is the second leading cause of death worldwide. In this study, anthraquinone derivatives (Compounds 1–5) were evaluated for their anti-cancer potential against various skin and breast cancer cell lines to assess whether these anthraquinone derivatives may serve as a lead for the augmentation of anti-cancer drug.

**Methods:**

Anthraquinone derivatives, 2-methyl-1,3,6-trihydroxy-9,10-anthraquinone-3-O-(6′-O-acetyl)-α-rhamnosyl(1 → 2)-β-glucoside (Comp 1), 2-methyl-1,3,6-trihydroxy-9,10-anthraquinone (Comp 2), and alizarin (Comp 3) were isolated from the dichloromethane fraction of the roots of *Rubia philippinensis*., whereas ethyl acetate fraction yielded xanthopurpurin (Comp 4) and lucidin-ω-methyl ether (Comp 5). Structures of all the isolated compounds were determined by spectral data analysis. All isolated compounds (Comp 1–5) were assessed for cytotoxicity by the 3-(4,5-dimethylthiazol-2-yl)-2,5-diphenyltetrazolium bromide (MTT) assay against four different cancer cell lines, i.e. human melanoma (SK-MEL-5), murine melanoma (B16F10), and human breast adenocarcinoma (MCF7 and MDA-MB-231).

**Results:**

Significant activity of the compounds 4 and 5 was observed against the breast cancer cell line MDA-MB-231 with IC_50_ values of 14.65 ± 1.45 and 13.03 ± 0.33 μM, respectively. Encouragingly, IC_50_ values of 67.89 ± 1.02 and 79.01 ± 0.03 μM against normal kidney epithelial cells (MDCK) were also obtained for compounds 4 and 5, respectively, which indicated very low toxicity and favorable selectivity indices for compounds 4 and 5 in the range of 1.85 to 3.95 and 2.11 to 6.06 against skin cancer cell lines (SK-MEL-5, and B16F10), and breast cancer cell lines (MCF7 and MDA-MB-231), respectively.

**Conclusion:**

Our results suggested that the compounds 4 (xanthopurpurin) and 5 (lucidin-ω-methyl ether) showed high selective toxicity towards breast cancer cells at lower concentrations without showing toxicity towards normal cells, thus could be of potential as new lead molecules in cancer treatment.

**Electronic supplementary material:**

The online version of this article (10.1186/s12906-018-2253-2) contains supplementary material, which is available to authorized users.

## Background

Cancer is one of the most frequently occurring diseases and is the second leading cause of death worldwide, while chemotherapy is most extensively used among a wide range of anti-cancer therapies, and its high toxicity, being expensive as well as activating alternative cell signaling pathways are limiting its applications [1]. For centuries to date, being safe, low cost and easily accessible, medicinal herbs are viewed as the main sources of new drugs to treat cancer worldwide while various pharmacological studies continue to validate their uses [1]. Moreover, herbal medicines are widely assumed in complementary and alternative medicine especially in cancer patients with poor socioeconomic condition. Mounting evidences suggest that plants possessing anticancer properties, such as *Soymida fembrifuga* (Miliaceae), *Tinospora cordifolia* (Menispermaceae), *Lavandula bipinnata* (Lamiaceae), *Helicteres isora* (Sterculiaceae), *Urtica membranacea* (Urticaceae), *Artemesia monosperma* (Asteraceae), and *Origanum dayi post* (Labiatae) etc., are the source of alternative medicine for cancer therapy in various regions of the globe [2–4]. However, a large number of plant species remain to be screened for their therapeutic potential; consequently, they can be used as a continual source of new medicines for present and future health problems of humans, including cancer.

*Rubia philippinensis* is a rambling and low climbing perennial herb that grows in the Southern part of Vietnam. Local communities have long utilized this medicinal plant to treat ordinary ailments such as wounds, inflammation, and skin infections. Previous investigations of the species have resulted in the purification of arborinane triterpenoids, which show promising effects on the prevention and treatment of atherosclerosis [5]. Additionally, rubiarbonone C, a popular chemical entity isolated from *R. philippinensis*, has been shown to inhibit abnormal proliferation and migration of vascular smooth muscle cells, which plays an important role in the pathophysiology of atherosclerosis. The mechanism by which rubiarbonone C regulates vascular remodeling was further clarified through focal adhesion kinase (FAK), MAPK, and STAT3 Tyr705 [6]. In searching for bioactive components from *R. philippinensis*, in this study, derivatives of anthraquinone were isolated as the major compounds.

Anthraquinones possessing three benzene rings represent a class of compounds belonging to quinone family. The divergence of the anthraquinone molecules relies on the nature and the setting of the substituents. Anthraquinones display a number of biological functions, including laxative [7], diuretic [8], phytoestrogen [9], anti-platelet [10], anti-fungal [11], anti-viral [12], and anti-cancer properties [13]. Moreover, they have a significant industrial potential of being used as textile dyes, food colorants and bugs repellents.

As a part of continuous attempts to probe the potential nature-derived drug templates for the treatment of cancer [5, 14], the current study delineates the isolation and characterization of five anthraquinone derivatives (compound 1–5) from *R. philippinensis*. These compounds were evaluated for their anti-cancer potential against various skin cancer cells (SK-MEL-5 and B16F10) and breast cancer cells (MCF7 and MDA-MB-231) to assess whether these anthraquinone derivatives may serve as a lead for the development of anti-cancer drugs.

## Methods

### Plant materials

Root samples of *Rubia philippinensis* were procured from Bidoup-Nui Ba National Park, Lamdong province, Vietnam and identified by the expert Dr. Phuong Thien Thuong at the Department of Pharmaceutical Analysis and Herbal Standardization, NIMM, Hanoi, Vietnam. An authenticated root voucher sample was deposited at the laboratory of the NIMM (VDL20140801) and at the Pharmacognosy Laboratory, College of Pharmacy, Chungnam National University (CNU1409), Daejeon, Korea.

### Extraction, isolation, and characterization of anthraquinone derivatives

Anthraquinones were isolated from the root samples of *R. philippinensis* by chromatographic techniques. In brief, the ethanol extract of *R. philippinensis* (150 g) was suspended in H_2_O (1.5 L) and sequentially partitioned with CH_2_Cl_2_ (2 L × 3) and EtOAc (2 L × 3) to yield the CH_2_Cl_2_ and EtOAc extracts. The CH_2_Cl_2_-soluble fraction (50 g) was loaded into silica gel VLC and eluted with *n*-hexane-EtOAc (20:1, 10:1, 5:1, 3:1, 2:1) and CHCl_3_-MeOH (8:1) to afford six fractions (D-1 → D-6). Fraction D-4 (6.1 g) was divided into 10 sub-fractions (D-4-1 → D-4-10) using MPLC with a step-wise gradient of Acetone-H_2_O (60:40, 72:28, 75:25, 95:5, 100:0, each 1.5 L). Xanthopurpurin (**4**) (*t*_R_ 33.5 min, 28 mg) and lucidin-ω-methyl ether (**5**) (*t*_R_ 36.0 min, 31 mg) were obtained from D-4-4 (320 mg) by HPLC eluting with MeCN-H_2_O (54.5:45.5, 4 mL/min, UV 360 nm). The EtOAc fraction (14.0 g) was subjected to silica gel VLC and eluted with *n*-hexane/EtOAc/MeOH (2:1:0.2) and CHCl_3_-MeOH (8:1, 5:1, 3:1, 0:1) to yield five fractions (EA-1 → EA-5). Eight sub-fractions (EA-1-1 → EA-1-8) were collected from fraction EA-1 (1.8 g) by utilizing MPLC, eluting with MeOH-H_2_O (10:90, 50:50, 67:33, 80:20, 100:0, each 500 mL). Alizarin (**3**) (*t*_R_ 44.0 min, 2 mg) was isolated from EA-1-5 (100 mg) by HPLC eluting with MeCN-H_2_O (44.5:55.5, 4 mL/min, UV 360 nm). Two sub-fractions EA-1-6 and EA-1-7 were combined (EA-1-6,7; 500 mg), then purified one more time by MPLC, eluted with Acetone-H_2_O (40:60, 60:40, 100:0, each 500 mL) to afford 2-methyl-1,3,6-trihydroxy-9,10-anthraquinone (**2**) as orange crystals (200 mg). Fraction EA-4 (2.6 g) was separated by MPLC applying mixtures of solvent MeOH-H_2_O (23:77, 37:63, 47:53, 52:48, 60:40, 67:33, 100:0, each 400 mL) to yield 11 sub-fractions (EA-4-1 → EA-4-11). 2-methyl-1,3,6-trihydroxy-9,10-anthraquinone-3-*O*-(6’-*O*-acetyl)-*α*-rhamnosyl(1 → 2)-*β*-glucoside (**1**) (*t*_R_ 40 min, 800 mg) was purified from sub-fractions EA-4-8 (706 mg) and EA-4-9 (410 mg) by HPLC utilizing MeOH-H_2_O (65:35, 6 mL/min, UV 254 nm).

2-methyl-1,3,6-trihydroxy-9,10-anthraquinone-3-*O*-(6’-*O*-acetyl)-*α*-rhamnosyl(1 → 2)-*β*-glucoside (**1**): yellow powder, ^1^H NMR (300 MHz, DMSO-*d*_6_): δ_H_ 13.28 (1H, s, OH-1), 8.08 (1H, d, *J* = 8.4 Hz, H-8), 7.45 (1H, d, *J* = 2.4 Hz, H-5), 7.40 (1H, s, H-4), 7.20 (1H, dd, *J* = 8.4, 2.4 Hz, H-7), 5.45 (1H, d, *J* = 6.9 Hz, Glu-H-1′), 5.28 (1H, d, *J* = 0.9 Hz, Rha-H-1″), 2.15 (3H, s, CH_3_–2), 1.93 (3H, s, OAc-6′), 1.09 (3H, d, *J* = 6.3 Hz, Rha-CH_3_–6″). ^13^C NMR (150 MHz, DMSO-*d*_6_) δ_C_ *Aglycone*: 164.2 (C-1), 120.5 (C-2), 160.0 (C-3), 105.2 (C-4), 135.4 (C-4a), 112.8 (C-5), 161.3 (C-6), 121.6 (C-7), 129.7 (C-8), 124.1 (C-8a), 186.3 (C-9), 110.7 (C-9a), 181.8 (C-10), 131.9 (C-10a), 8.7 (CH_3_–2). *Glucose*: 97.3 (C-1′), 76.3 (C-2′), 77.0 (C-3′), 70.0 (C-4′), 74.0 (C-5′), 63.3 (C-6′), 170.3 (OAc-6′), 20.4 (OAc-6″). *Rhamnose*: 100.2 (C-1″), 70.3 (C-2″), 70.5 (C-3″), 72.0 (C-4″), 68.5 (C-5″), 18.1 (C-6″).

2-methyl-1,3,6-trihydroxy-9,10-anthraquinone (**2**): orange crystal, ^1^H NMR (300 MHz, DMSO-*d*_6_): δ_H_ 13.31 (1H, s, OH-1), 8.05 (1H, d, *J* = 8.4 Hz, H-8), 7.43 (1H, d, *J* = 2.4 Hz, H-5), 7.20 (1H, s, H-4), 7.20 (1H, dd, *J* = 8.4, 2.4 Hz, H-7), 2.05 (3H, s, CH_3_–2). ^13^C NMR (75 MHz, DMSO-*d*_6_) δ_C_ 162.3 (C-1), 117.4 (C-2), 163.1 (C-3), 107.1 (C-4), 135.2 (C-4a), 112.5 (C-5), 162.1 (C-6), 121.3 (C-7), 129.4 (C-8), 124.7 (C-8a), 185.8 (C-9), 108.6 (C-9a), 182.0 (C-10), 131.8 (C-10a), 8.1 (CH_3_–2).

Alizarin (**3**): brownish red powder, ^1^H NMR (300 MHz, DMSO-*d*_6_) δ_H_ 7.51 (1H, d, *J* = 8.1, H-4), 7.18 (1H, d, *J* = 8.1, H-3). ^13^C NMR (150 MHz, DMSO-*d*_6_) δ_C_ 151.0 (C-1), 153.3 (C-2), 120.8 (C-3), 121.3 (C-4), 126.5 (C-5), 134.0 (C-6), 135.1 (C-7), 126.7 (C-8), 188.8 (C-9), 180.5 (C-10), 123.5 (C-4a), 132.9 (C-10a), 133.7 (C-8a), 116.2 (C-9a).

Xanthopurpurin (**4**): orange powder, ^1^H NMR (300 MHz, DMSO-*d*_6_) δ_H_ 7.79 (1H, d, *J* = 2.1, H-4), 7.27 (1H, d, *J* = 2.1, H-3). ^13^C NMR (75 MHz, DMSO-*d*_6_) δ_C_ 164.7 (C-1), 107.6 (C-2), 165.5 (C-3), 108.4 (C-4), 126.7 (C-5), 134.5 (C-6), 134.3 (C-7), 126.2 (C-8), 185.6 (C-9), 181.6 (C-10), 134.7 (C-4a), 132.7 (C-10a), 132.8 (C-8a), 109.1 (C-9a).

Lucidin-ω-methyl ether (**5**): orange powder, ^1^H NMR (300 MHz, CDCl_3_) δ_H_ 13.24 (1H, s, OH-1), 4.89 (2H, s, CH_2_OCH_3_–2), 3.55 (3H, s, CH_2_OCH_3_–2). ^13^C NMR (75 MHz, CDCl_3_) δ_C_ 162.0 (C-1), 114.5 (C-2), 164.2 (C-3), 109.7 (C-4), 127.5 (C-5), 134.3 (C-6), 134.2 (C-7), 126.8 (C-8), 187.0 (C-9), 182.3 (C-10), 133.6 (C-4a), 134.2 (C-10a), 133.6 (C-8a), 109.9 (C-9a), 69.0 (CH_2_OCH_3_–2), 59.5 (CH_2_OCH_3_–2).

### Cell culture and cell viability assay

The potential cytotoxicity of the isolated anthraquinone derivatives was studied against various cancer cell lines, including SK-MEL-5 (human melanoma), B16F10 (murine melanoma) MCF7 (human breast adenocarcinoma), and MDA-MB-231 (human breast adenocarcinoma) and the normal cell line MDCK (normal kidney epithelial) using the MTT assay [15]. All cell lines were cultured in DMEM medium supplemented with 10% foetal bovine serum (FBS) and streptomycin–penicillin (100 μg/ml each; Hyclone) in a 5% CO_2_ humidified incubator. An MTT assay was employed to determine the percentage of the viability of various cancer cells as well as MDCK cells. All cells were first cultured in 96-well plates (1 × 10^5^ cells/mL for all cancerous cells and 5 × 10^5^ cells/mL for MDCK cells) for 24 h, and treated with indicated concentration of isolated compounds (6.25–100 μM for cancerous cells and 6.25–400 μM for MDCK cells). Various dilutions of stock culture were made in the culture medium to get the final concentration of the sample with a 0.1% of DMSO concentration, including the control. After 24 h incubation, MTT reagent was added to each well and the plate was incubated at 37 °C for 1 h. After removing the medium, the plate was washed twice with PBS (pH 7.4). The intracellular insoluble formazan was dissolved in 100% DMSO. A microplate reader was used to measure the absorbance of each cell line at 570 nm, and the percentage of cell viability was calculated. The absorbance value for the average of wells of cells treated with each test sample concentration was expressed as a percentage of this control and the IC_50_ values for each sample on each cell line were calculated. The anti-cancer drug oxaloplatin was used as a positive control.

### Statistical analysis

All the results were presented as the mean ± SD following the analysis of one-way ANOVA. A value of *p* < 0.05 was recognized as significant for the differences. An SPSS version of Windows’ (Chicago, Illinois, USA) was performed for all the analyses.

## Results

### Identification and characterization of anthraquinone derivatives (Fig. 1)

The ^1^H NMR data of compound 1 displayed signals of the anthraquinone aglycone, including one aromatic singlet proton δ_H_ 7.40 (1H, s, H-4), one ABX ring system δ_H_ 8.08 (1H, d, *J* = 8.4 Hz, H-8), 7.45 (1H, d, *J* = 2.4 Hz, H-5), 7.20 (1H, dd, *J* = 8.4, 2.4 Hz, H-7), and one singlet methyl δ_H_ 2.15. The glycosidic linkage, meanwhile, contained resonances of two anomeric protons of the sugar moiety at δ_H_ 5.45 (glucose), δ_H_ 5.28 (rhamnose), one secondary methyl (δ_H_ 1.09, rhamnose), and one acetyl group (δ_H_ 1.93). The ^13^C NMR data showed 14 signals of a typical anthraquinone, including two ketones (δ_C_ 186.3, 181.8), and resonances for glucose (δ_C_ 97.3, 76.3, 77.0, 70.0, 74.0, 63.3), acetoxy (δ_C_ 170.3, 20.4), and rhamnose (δ_C_ 100.2, 70.3, 70.5, 72.0, 68.5, 18.1) moieties. On the basis of NMR spectroscopic data analyses, the compound was identified as 2-methyl-1,3,6-trihydroxy-9,10-anthraquinone-3-*O*-(6’-*O*-acetyl)-*α*-rhamnosyl(1 → 2)-*β*-glucoside.

**Fig. 1:**
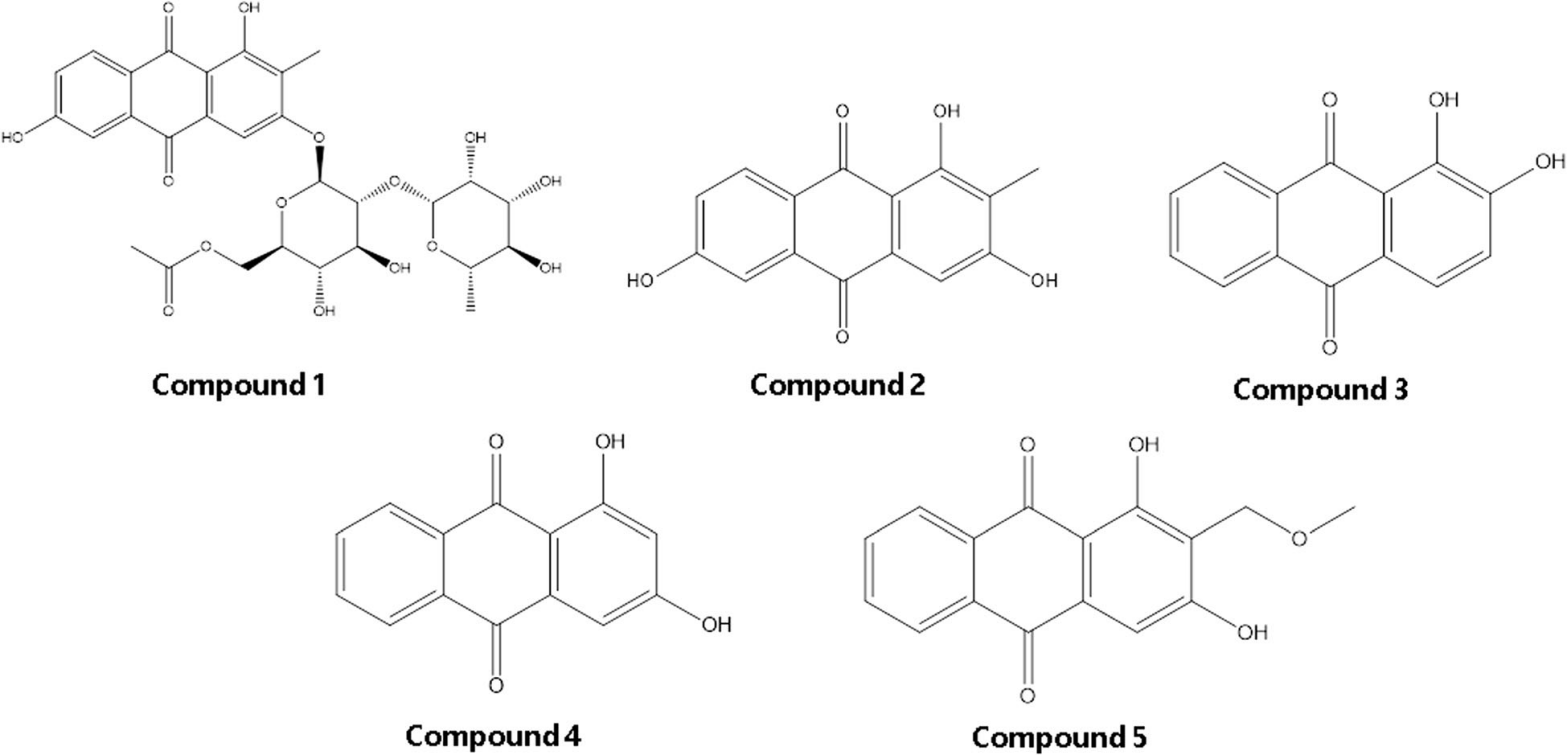
Chemical structures of anthraquinone derivatives, 2-methyl-1,3,6-trihydroxy-9,10-anthraquinone 3-*O*-(6ʹ-*O*-acetyl)-α-rhamnosyl(1 → 2)-β-glucoside (compound 1), 2-methyl-1,3,6-trihydroxy-9,10-anthraquinone (compound 2), alizarin (compound 3), xanthopurpurin (compound 4), and lucidin-ω-methyl ether (compound 5) isolated from *R. philippinensis*

Similar to compound 1, compound 2 also showed resonances of one aromatic singlet proton, one ABX ring system, and one singlet methyl at δ_H_ 7.20 (1H, s, H-4); [δ_H_ 8.05 (1H, d, *J* = 8.4 Hz, H-8; 7.43 (1H, d, *J* = 2.4 Hz, H-5; 7.20 (1H, dd, *J* = 8.4, 2.4 Hz, H-7)]; and δ_H_ 2.05 (3H, s, CH_3_–2), respectively in ^1^H NMR spectrum. On the other hand, the skeleton of 14 carbon signals along with two ketonic carbonyls (δ_C_ 185.8, 182.0) and one methyl functionality (δ_C_ 8.1) was representative of ^13^C NMR data of an anthraquinone. The 1D NMR of compound 2 resemble closely to those of compound 1, except for the absence of signals belonging to sugar units. In comparison with reference values, compound 2 was determined as 2-methyl-1,3,6-trihydroxy-9,10-anthraquinone. Compound 3, 4, and 5 are also anthraquinone derivatives and their structures were elucidated as alizarin, xanthopurpurin, and lucidin-ω-methyl ether, respectively, based on the NMR data analysis. NMR data of all anthraquinone has been provided in Additional file 1: Figures S1-S5).

### Cytotoxicity of anthraquinone derivatives

All compounds were tested for cytotoxicity by MTT assay on cell lines SK-MEL-5, B16F10, MCF7, MDA-MB-231, and MDCK cells as a normal cell line, which showed significant cytotoxicity (Table 1, Additional file 1: Figures S6-S10). Our results showed that the IC_50_ values for cancer cell lines treated ranged from 48.68 ± 0.10 to 91.04 ± 1.88 μM for compound 1; 46.75 ± 1.39 to 79.96 ± 1.14 μM for compound 2; 48.64 ± 0.33 to 98.79 ± 2.10 μM for compound 3, 14.65 ± 1.45 to 23.71 ± 1.71 μM for compound 4, and 13.03 ± 0.33 to 42.79 ± 1.32 μM for compound 5. Regarding the normal cell line MDCK cells, the IC_50_ values were 192.34 ± 0.49, 168.76 ± 0.61, 199.32 ± 1.88, 67.89 ± 1.02 and 79.01 ± 0.03 μM for compounds 1, 2, 3, 4, and 5, respectively. Interestingly, among all the compounds, compounds 4 and 5 showed strong cytotoxicity towards breast cancer cells (MCF7 and MDA-MB-231) than skin cancer cells (SK-MEL-5 and B16F10) with IC_50_ value of 15.75 ± 1.00 and 24.10 ± 1.06 for MCF7 as well as 14.65 ± 1.45 and 13.03 ± 0.33 for MDA-MB-231, respectively.

**Table 1:** IC_50_ values of anthraquinone derivatives (compound 1–5) on various skin cancer cells (SK-MEL5 and B16F10) and breast cancer cells (MCF7 and MBA-MD-231)

Compounds	IC_50_ (μM)^a^				
	SK-MEL-5	B16F10	MCF7	MDA-MB-231	MDCK
1	91.04 ± 1.88	48.68 ± 0.10	65.48 ± 1.10	49.44 ± 0.78	192.34 ± 0.49
2	46.75 ± 1.39	77.88 ± 0.34	79.96 ± 1.14	59.22 ± 0.40	168.76 ± 0.61
3	53.08 ± 0.30	98.79 ± 2.10	49.17 ± 0.85	48.64 ± 0.33	199.32 ± 1.88
4	21.35 ± 0.99	23.71 ± 1.71	15.75 ± 1.00	14.65 ± 1.45	67.89 ± 1.02
5	42.79 ± 1.32	29.48 ± 2.61	24.10 ± 1.06	13.03 ± 0.33	79.01 ± 0.03
Oxaloplatin	14.25 ± 1.02	10.51 ± 0.92	8.59 ± 1.22	7.95 ± 1.92	24.02 ± 1.04

^a^The values are mean ± standard deviation. IC_50_ (concentration inhibiting 50% growth). SK-MEL-5 (human melanoma); B16F10 (murine melanoma); MCF-7 (human breast adenocarcinoma); MDA-MB-231 (human breast adenocarcinoma), MDCK (normal kidney epithelial cells)

In addition, compound 4 and 5 were more cytotoxic to MDA-MB-231 cancer cell line (IC_50_ = 14.65 ± 1.45 and 13.03 ± 0.33 μM, respectively) than to normal cells (IC_50_ = 67.89 ± 1.02 and 79.01 ± 0.03 μM (Table 1), respectively with their respective selectivity indices of 4.63 and 6.06 (Table 2).

**Table 2:** Selectivity of the cytotoxicity of anthraquinone derivatives (compound 1–5) to various cancer cells as compared with MDCK cells

Compounds	IC_50_ (μM)^a^			
	SK-MEL-5	B16F10	MCF7	MDA-MB-231
1	2.11	3.95	2.94	3.89
2	3.61	2.17	2.11	2.85
3	3.76	2.02	4.05	4.10
4	3.18	2.86	4.31	4.63
5	1.85	2.68	3.28	6.06

^a^The selectivity index is the ratio of the IC_50_ values of the treatments on MDCK cells to those in the cancer cell lines. SK-MEL-5 (human melanoma); B16F10 (murine melanoma); MCF-7 (human breast adenocarcinoma); MDA-MB-231 (human breast adenocarcinoma)

Table 2 shows the selectivity indices of the isolated compounds tested against the various cancer cell lines and the non-tumor cell line (MDCK). In the current study, treatments with compound 4 and 5 afforded the highest selectivity indices in breast cancer cell than skin cancer cells. Compound 4 showed the selectivity indices as 4.31 and 4.63 whereas compound 5 showed 3.28 and 6.06 in MCF7 and MDA-MB-231 cells, respectively (Table 2).

## Discussion

A number of natural compounds have been isolated from different plant sources which have shown enormous biological potential [16–19]. In this study, five anthraquinone derivatives, such as 2-methyl-1,3,6-trihydroxy-9,10-anthraquinone 3-*O*-(6'-*O*-acetyl)-α-rhamnosyl(1 → 2)-β-glucoside (compound 1), 2-methyl-1,3,6-trihydroxy-9,10-anthraquinone (compound 2), alizarin (compound 3), xanthopurpurin (compound 4), and lucidin-ω-methyl ether (compound 5) were isolated from the root of *R. philippinensis*., and were characterized based on the spectral data analysis [16–19].

These anthraquinone derivatives showed significant anticancer potential as confirmed by their cytotoxicity effects against various cancer cell lines, such as cell lines SK-MEL-5, B16F10, MCF7, MDA-MB-231, including normal MDCK cell line. However, according to American National Center Institute, extract/compounds with IC_50_ values lower than 30 μM against experimental cancer cell lines constitute promising anticancer agents for drug development [20]. Therefore, compound 4 and 5 showed IC_50_ values greater than 30 μM against all cell lines tested, and were more cytotoxic to normal line to which the cancer cell lines. Moreover, among the testest compounds, anthraquinone derivatives xanthopurpurin (compound 4), and lucidin-ω-methyl ether (compound 5) showed highest selectivity indices in breast cancer cell than skin cancer cells.

Mounting evidences have considered that a value greater than or equals to 2.0 is an interesting selectivity index [21]. This value means that the compound is more than twice more cytotoxic to the cancer cell line as compared with the normal cell line [21]. These findings demonstrated that compound 4 and 5 can be considered promising lead molecules for the development of anticancer drugs, especially for breast cancer, because they provided indices value greater than 2.

## Conclusions

It is very important to consider natural compounds as a chemotherapeutic agent for cancer which have minimum or no side effects on normal body cells of patients. To achieve this goal among various ways, one of the way is by employing lower doses of drug at which drug shows highly potent activity as well as exhibits high degree of selectivity. In this study, we presented the cytotoxicity potential of five anthraquinone derivatives isolated from the roots of *Rubia philippinensis*. The results of in vitro studies demonstrate the ability of the compounds 4 (xanthopurpurin) and 5 (lucidin-ω-methyl ether) for high selective toxicity at lower concentrations (Table 1) without showing toxicity towards normal cells, confirming that compounds 4 and 5 may have the potentiality to be developed as anticancer drugs, especially for breast cancer. Further research strategies should investigate cytotoxic potential of compound 4 and 5 against multifactorial drug-resistant cancers for their pharmaceutical formulations.

## Additional file

Additional file 1: Supplementary data contain ten supplementary figures. Among them Figures S1-S5 represent proton and carbon NMR data of the isolated compounds, whereas Figures S6-S10 represent cytotoxic effects of isolated compounds (1–5) against MDCK, SK-MEL-5, B16F10, MCF7, and MDA-MB-231 cell lines. (DOC 899 kb)

## Abbreviations

ANOVA: Analysis of variance
DMEM: Dulbecco'’s Modified Eagle'’s Medium
DMSO: Dimethyl sulfoxide
FAK: Focal adhesion kinase
FBS: Foetal bovine serum
HPLC: High-performance liquid chromatography
MAPK: Mitogen-activated protein kinase
MPLC: Medium pressure liquid chromatography
MTT: 3-(4,5-dimethylthiazol-2-yl)-2,5-diphenyltetrazolium bromide
NMR: Nuclear magnetic resonance
SD: Standard deviation
UV: Ultraviolet
VLC: Vacuum liquid chromatography
