# Supplementary material for: Cytotoxic properties of the anthraquinone derivatives isolated from the roots of Rubia philippinensis
Source: BMC Complement Altern Med. 2018 Jul 3;18:200. doi: 10.1186/s12906-018-2253-2 (PMC6029275; doi:10.1186/s12906-018-2253-2)
Supplement: Supplementary file 1 — Supplementary data contain ten supplementary figures. Among them Figures S1-S5 represent proton and carbon NMR data of the isolated compounds, whereas Figures S6-S10 represent cytotoxic effects of isolated compounds (1–5) against MDCK, SK-MEL-5, B16F10, MCF7, and MDA-MB-231 cell lines. (DOC 899 kb) [file 12906_2018_2253_MOESM1_ESM.doc]

**Cytotoxic properties of the anthraquinone derivatives isolated from the** **roots of *Rubia philippinensis***

**Vivek K. Bajpai^1^, Md Badrul Alam^2,3^, Khong Trong Quan^4^, Hee-Jeong Choi^2^, Hongyan An^2^, Mi-Kyoung Ju^2^, Sang-Han Lee^2,3*^, Yun Suk Huh^5^, Young-Kyu Han^1,*^, MinKyun Na^4*^**

^1^Department of Energy and Materials Engineering, Dongguk University-Seoul, Seoul 04620, Republic of Korea

^2^Department of Food Science and Biotechnology, Graduate School, Kyungpook National University, Daegu 41566, Republic of Korea

^3^Food and Bio-Industry Research Institute, Kyungpook National University, Daegu 41566, Republic of Korea

^4^College of Pharmacy, Chungnam National University, Daejeon 34134, Republic of Korea

^5^Department of Biological Engineering, Biohybrid Systems Research Center (BSRC), Inha University, 100 Inha-ro, Nam-gu, Incheon 22212, Republic of Korea

**Running head:** Cytotoxic anthraquinone derivatives from *R. philippinensis*

***Corresponding authors:**

**Dr. Sang-Han Lee**; Email: [sang@knu.ac.kr](mailto:sang@knu.ac.kr)

**Dr. MinKyun Na**; E-mail: [mkna@cnu.ac.kr](mailto:mkna@cnu.ac.kr)

**Dr. Young-Kyu Han; E-mail:** [ykenergy@dongguk.edu](mailto:ykenergy@dongguk.edu)

**Fig. S1 (A).** ^1^H NMR spectrum of compound **1** (300 MHz, DMSO)

**Fig. S1 (B).** ^13^C NMR spectrum of compound **1** (75 MHz, DMSO)

**Fig. S1 (C).** ^13^C NMR spectrum of compound **1** (150 MHz, DMSO)

**Fig. S2 (A).** ^1^H NMR spectrum of compound **2** (300 MHz, DMSO)

**Fig. S2 (B).** ^13^C NMR spectrum of compound **2** (75 MHz, DMSO)

**Fig. S3 (A).** ^1^H NMR spectrum of compound **3** (300 MHz, DMSO)

**Fig. S3 (B).** ^13^C NMR spectrum of compound **3** (150 MHz, DMSO)

**Fig. S4 (A).** ^1^H NMR spectrum of compound **4** (300 MHz, DMSO)

**Fig. S4 (B).** ^13^C NMR spectrum of compound **4** (75 MHz, DMSO)

**Fig. S5 (A).** ^1^H NMR spectrum of compound **5** (300 MHz, CDCl_3_)

**Fig. S5 (B).** ^13^C NMR spectrum of compound **5** (75 MHz, CDCl_3_)


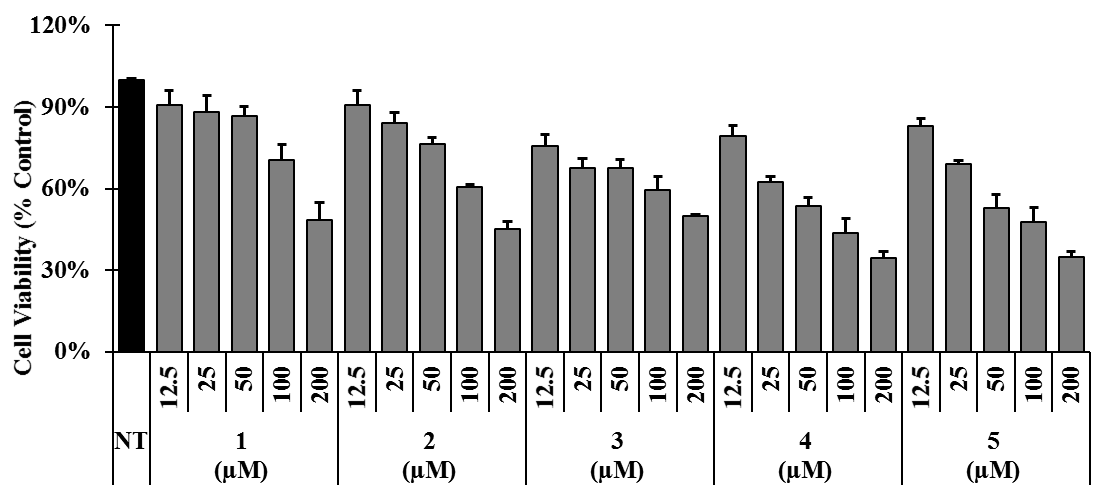


**Fig. S6** Effect of anthraquinone derivatives (compound 1-5) on the viability of MDCK cells. Cells were treated with indicated concentration of compounds 1-5 for 24 h and cell viability was determined by MTT assay. Values are expressed as the mean ± SD (n=3).


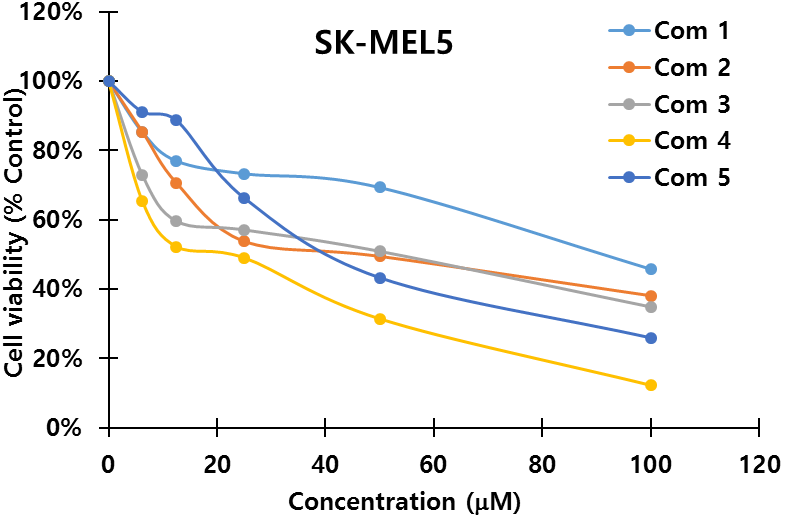


**Fig. S7** Effect of anthraquinone derivatives (compound 1-5) on the viability of SK-MEL-5 cells. Cells were treated with indicated concentration of compounds 1-5 for 24 h and cell viability was determined by MTT assay. Values are expressed as the mean ± SD (n=3).


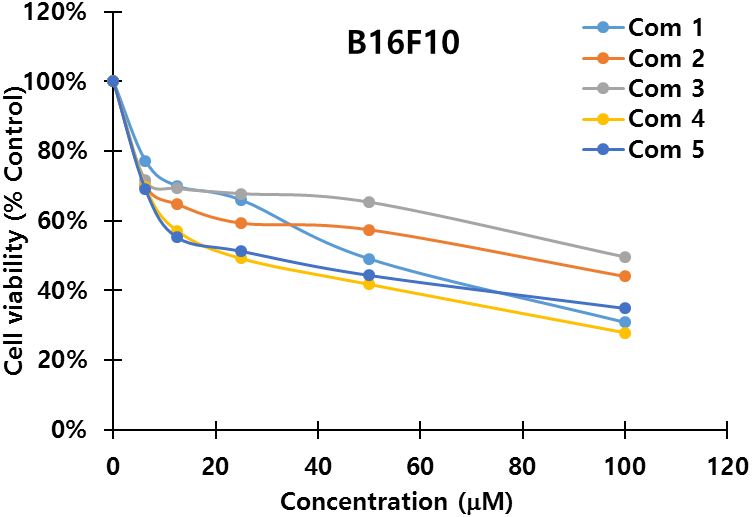


**Fig. S8** Effect of anthraquinone derivatives (compound 1-5) on the viability of B16F10 cells. Cells were treated with indicated concentration of compounds 1-5 for 24 h and cell viability was determined by MTT assay. Values are expressed as the mean ± SD (n=3).


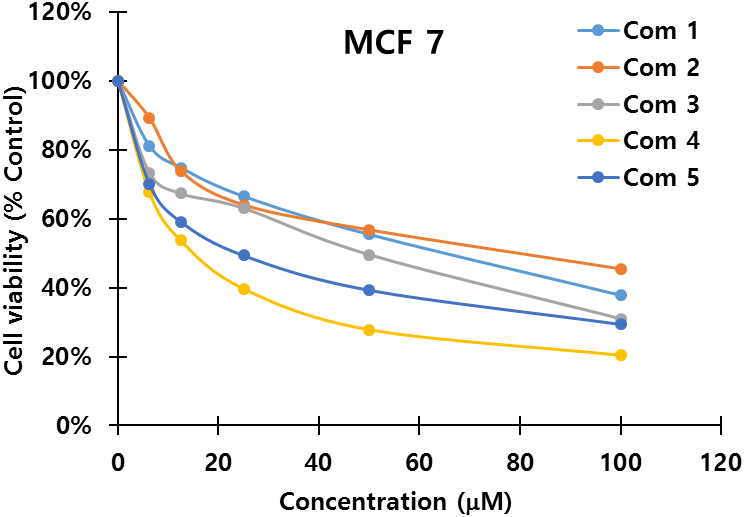


**Fig. S9** Effect of anthraquinone derivatives (compound 1-5) on the viability of MCF7 cells. Cells were treated with indicated concentration of compounds 1-5 for 24 h and cell viability was determined by MTT assay. Values are expressed as the mean ± SD (n=3).


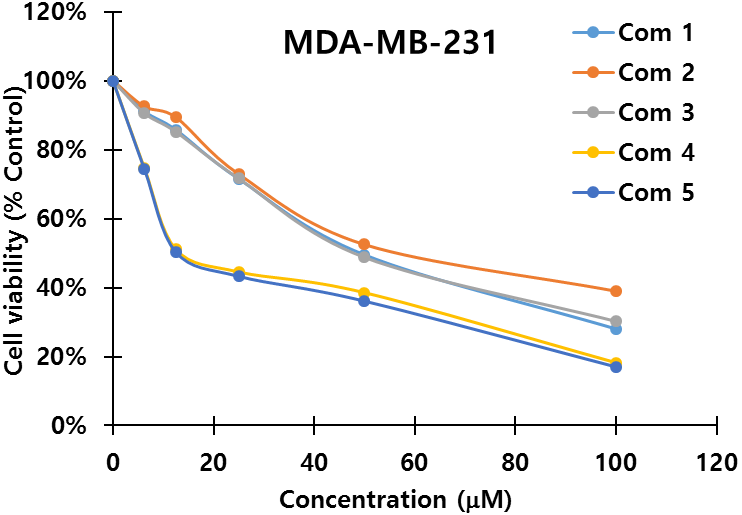


**Fig. S10** Effect of anthraquinone derivatives (compound 1-5) on the viability of MDA-MB-231 cells. Cells were treated with indicated concentration of compounds 1-5 for 24 h and cell viability was determined by MTT assay. Values are expressed as the mean ± SD (n=3).
